# Ethanol-Mediated 2D Growth of Cu_2_O Nanoarchitectures on Nanoporous Cu Templates in Anhydrous Ethanol

**DOI:** 10.3390/nano8010018

**Published:** 2017-12-31

**Authors:** Zhenhua Dan, Jiafei Lu, Feng Li, Fengxiang Qin, Hui Chang

**Affiliations:** 1 Tech Institute for Advanced Materials and College of Materials Science and Engineering, Nanjing Tech University, Nanjing 210009, China; dan9506@gmail.com (J.L.); fengli@njtech.edu.cn (F.L.); ch2006@njtech.edu.cn (H.C.); 2 The Synergetic Innovation Center for Advanced Materials, Nanjing Tech University, Nanjing 210009, China; 3 School of Materials and Science and Engineering, Nanjing University of Science and Technology, Nanjing 210094, China

**Keywords:** Cu_2_O, nanobelts, nanopetals, nanoporous copper templates, anhydrous ethanol

## Abstract

Two types of cupric oxide (Cu_2_O) nanoarchitectures (nanobelts and nanopetal networks) have been achieved via immersion nanoporous copper (NPC) templates in anhydrous ethanol. NPC templates with different defect densities have been prepared by dealloying amorphous Ti_60_Cu_40_ ribbons in a mixture solution of hydrofluoric acid and polyvinylpyrrolidone (PVP) with different ratios of HF/PVP. Both a water molecule reactant acting as OH^−^ reservoir and the ethanol molecule serving as stabilizing or capping reagent for inhibiting the random growth of Cu_2_Oplayed a role of the formation of 2-dimensional Cu_2_O nanoarchitectures. Cu_2_O nanobelts are preferred to form in anhydrous ethanol on the NPC templates from Ti_60_Cu_40_ ribbons dealloying in the solution with low HF concentration and small addition of PVP; and Cu_2_O nanopetals are tended to grow in anhydrous ethanol from the NPC templates from Ti_60_Cu_40_ ribbons dealloying in the solution with high HF concentration and large addition of PVP. With increasing the immersion time in anhydrous ethanol, Cu_2_O nanopetals united together to create porous networks about 300 nm in thickness. The defect sites (i.e., twin boundary) on nanoporous Cu ligaments preferentially served as nucleation sites for Cu_2_O nanocrystals, and the higher defect density leads to the formation of uniform Cu_2_O layer. Synergistic effect of initial microstructure of NPC templates and stabilizing agent of ethanol molecule results in different Cu_2_O nanoarchitectures.

## 1. Introduction

Cuprous oxide (Cu_2_O) is a p-type semiconductor with a direct band gap of 2.17 eV. Owing to the quantum size effects, Cu_2_O nanoparticles show many superior optical, electrical, and photo-electrochemical properties [1,2,3,4,5,6,7,8,9]. Because the shape and size of metal oxides directly affect their physical and chemical properties, many efforts have been devoted to the synthesis of uniform cuprous oxide with various structure and morphologies during the past decades [1,2,3,4]. Hydrazine [5], sodium borohydride [6], ethylene glycol [7], glucose [8], 2,5-dimethoxyaniline [9] or polyvinylpyrrolidone (PVP) [4] is frequently used to synthesize Cu_2_O particles, but rare report is found using water as a reducing agent. Many shapes of Cu_2_O nanostructures have been done last several decades, such as nanocubes via a surfactant-assisted solution-phase route [10,11], nanocages by PVP-mediated polyol process [12,13,14], microspheres by one-pot solvent-thermal method [15], nanorods by solvent-thermal method [16,17], nanowires by a hydrothermal approach [9,18], nanooctahedra through a reducing complex solution approach [19,20], nanoflowers via a polymer-assisted solution-phase route [6], double tower-tip-like Cu_2_O nanostructures in water/oil microemulsion [20], nanobranches by an additives-assisted electrochemical deposition [21,22,23,24,25], etc. Proper combination of the precursor, template, solvent, reductants helps to tailor the crystal habit via preferential adsorption of reactants, nucleation and growth of nanoarchitectures. Nanoporous noble metals with a high surface area and conductivity, such as Pt, Pd, Au, Ag, [26,27,28] have been fabricated via dealloying process. In order to reduce the cost of the nanoporous noble metals, many cost-effective metals with porous structures, such as Ni and Cu [29,30,31], have also been fabricated. The relative rough morphology with large nanopores and coarse ligaments restricts the potential application of nanoporous Cu in the catalytic and energy fields. Many efforts have been contributed to refine nanoporous Cu by alloying of third elements with a low surface diffusivity [30,32] and an introduction of PVP macromolecule into dealloying solution [33,34] down to few tenth nanometers through controlling the surface diffusion and the rearrangement of Cu adatoms. Cu_2_O layer readily forms due to the existence of the highly active sites on the Cu ligaments and exhibits superior performances of photodegradation [35] and Li-ion battery [36]. However, the other-shape Cu_2_O formation on nanoporous Cu has been rarely reported. 

In the present study, the effect of the combination of the nanoporous templates and ethanol as a stabilizing agent on the formation of the different shapes of Cu_2_O nanostructures has been investigated. The formation mechanism of Cu_2_O nanobelts and nanopetal networks was discussed on the basis of the growth of Cu_2_O nanobelts in anhydrous ethanol from analysis of X-ray diffractometry, scanning electron microscopy, transmission electron microscopy, X-ray photoelectron spectroscopy.

## 2. Materials and Methods

Nanoporous Cu (NPC) templates were prepared by dealloying the melt-spun Ti_60_Cu_40_ ribbons with a width of 2 mm and thickness of ~25 μm in the mixture solution of 0.03 M HF and 0.01 M PVP (1 g/L), and 0.65 MHF and 0.1 MPVP (10 g/L). The detail information of the experiments has been described previously [29,34]. The molecular weight of polyvinylpyrrolidone (PVP: (C_6_H_9_NO)*_n_*, Sigma-Aldrich Co. Ltd., Shanghai, China) was 55,000 g·mol^−1^ in the present experiments. Nanoporous Cu templates (NPC templates) were then immersed into purchased chemical reagent anhydrous ethanol (purity > 99.5 mass%, AR, Sinopharm Chemical Reagent Co. Ltd., Shanghai, China) for 24, 120 h. The water concentration of the anhydrous ethanol was about 0.5 mass%. The surface morphology of Cu_2_O nanoarchitectures were observed by scanning electron microscope (SEM, JEOL, FIB4610, JEOL Ltd., Tokyo, Japan). The as-spun ribbons, dealloyed ribbons and NPC templates after immersion in anhydrous ethanol were characterized by X-ray diffractometor (XRD, Rigaku, RINT-4200, Rigaku Co., Tokyo, Japan). The microstructure of NPC templates and the Cu_2_O nanostructures was observed by a transmission electron microscope (TEM, JEOL, HC2100, JEOL Ltd., Tokyo, Japan) and a high-resolution transmission electron microscope (HRTEM, JEOL, ARM200, JEOL Ltd., Tokyo, Japan). The TEM samples were prepared by focused ion beam milling (FIB, JEOL, Dual beam FIB 4610, JEOL Ltd., Tokyo, Japan) after coating with a protective carbon layer and a W deposition layer to lessen the damage of the surface layer. The surface chemical state was analyzed by an X-ray photoelectron spectroscope (XPS, Shimadzu Kratos, AXIS-Ultra DLD, Tokyo, Japan) with a monochromatized Al Kα radiation source (1486.6 eV).

## 3. Results and Discussion

### 3.1. 2D Growth of Cu_2_O Nanobelts in Anhydrous Ethanol on NPC Templates from Ti_60_Cu_40_ Ribbons Dealloying in 0.03 M HF and 0.01 M PVP Solution

**Figure 1:**
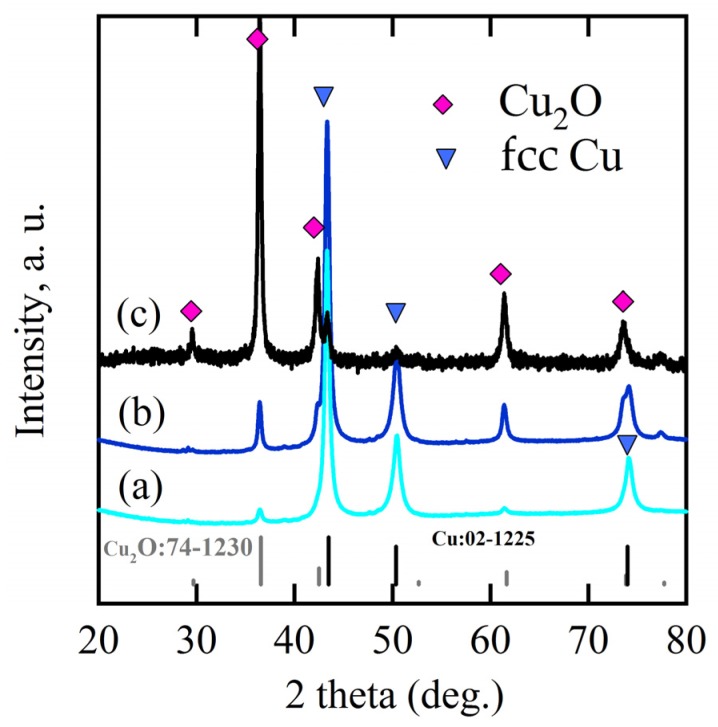
XRD pattern of Ti_60_Cu_40_ ribbon dealloyed in 0.03 M HF + 0.01 M PVP solution after immersion of 120 h in anhydrous ethanol (**a**) and XRD pattern of amorphous Ti_60_Cu_40_ ribbons after dealloying in 0.65 M HF + 0.1 M PVP after immersion of 24 h (**b**) and 120 h in anhydrous ethanol (**c**).

**Figure 2:**
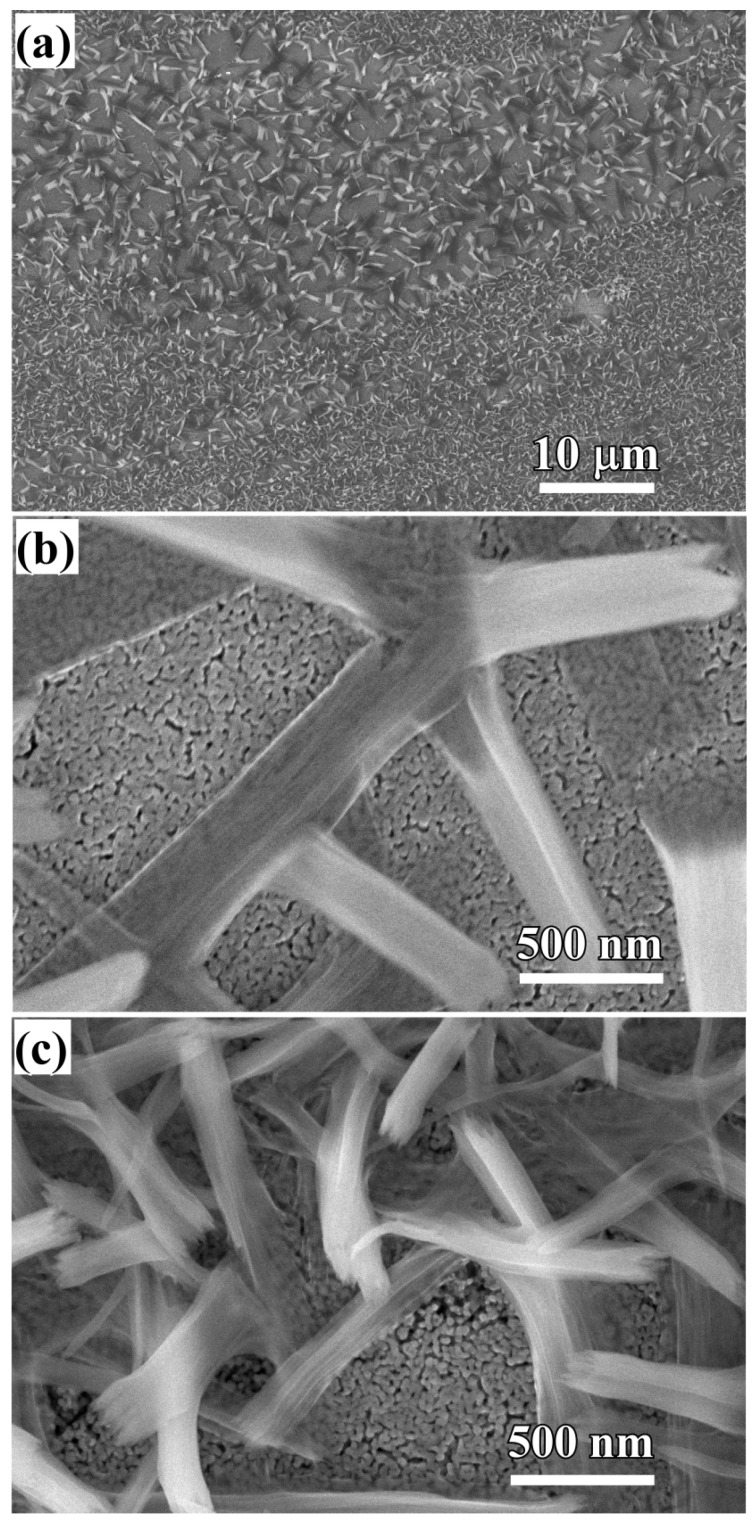
SEM morphology of Ti_60_Cu_40_ ribbon dealloyed in 0.03 M HF + 0.01 M PVP solution after immersion of 120 h in anhydrous ethanol (**a**) and its magnified images at middle region (**b**) and the edge region (**c**).

**Figure 3:**
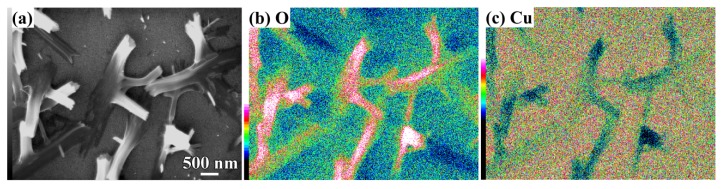
SEM image (**a**) and its elemental distribution of O (**b**) and Cu (**c**) of Ti_60_Cu_40_ ribbon dealloyed in 0.03 M HF + 0.01 M PVP solution after immersion of 120 h in anhydrous ethanol.

The crystalline state of the chemical composition of NPC templates and the characterization of the initial Ti_60_Cu_40_ precursor alloys have been reported previously [29,34]. The crystalline state of surface oxides on NPC templates after immersion of 120 h in anhydrous ethanol were confirmed by XRD (Figure 1a). The black and gray vertical lines stand for the peak position and intensity of Cu (JCPDF card No.: 02-1225) and Cu_2_O (JCPDF card No.: 74-1230). After immersing NPC templates in anhydrous ethanol for 120 h, two main diffraction peaks centered at 2θ = 36.5°, 61.5° were assigned to Cu_2_O (111) (220), and another diffraction peak from Cu_2_O (200) was overlapped by the diffraction peaks of fcc Cu around 2θ = 42°. The XRD data demonstrated that the Cu_2_O architectures formed after a free immersion in anhydrous ethanol for 120 h were crystalline. Meanwhile, three diffraction peaks from NPC templates were also detected and assigned to Cu (111), (200) and (220), which might be due to the partial coverage of Cu_2_O. As shown in Figure 2a, many belt-shaped species formed and covered partially the surface of NPC templates although the distribution was not uniform. The immersion products in this case are hereafter referred as nanobelts. The magnified morphology shows that the nanobelts with different sizes formed in the different region. In the middle regions, the belts have a characteristic width size of about 480 nm and the branch length of about few micrometers. The thickness of the belts isvery small since many of them are transparent under the secondary electron observation. Most of nanobelts have several branches shown in Figure 2b. On the other regions, the nanobelts with narrower branches with a width of about 160 nm became interlaced together and covered more than that in the middle region. The end regions of many belts exhibit serrated characteristics which are considered to be resulted from the fracture of the long belts due to removal of the residual chemicals via washing of water and ethanol. To some extent, this is also a supportive evidence for the nanobelts with a very thin thickness. It is worth noting that the underlying nanoporous structure covered by the larger nanobelts has a finer nanoporous structure in comparison to that covered by the finer nanobelts. This fact might be linked with the different distribution of the defects in different nanoporous structures. The elemental mapping profiles of the nanobelts in Figure 3 shows that the nanobelts consisted of O and Cu. The oxygen concentration of the nanobelts was higher than that of the underlying NPC templates. The chemical composition of the nanobelts can be confirmed to be Cu_2_O on the basis of the corresponding XRD pattern in Figure 1 and Figure 3. In Figure 3a, the main branches grew up from NPC templates and some small secondary branches grew from the main branches. 

**Figure 4:**
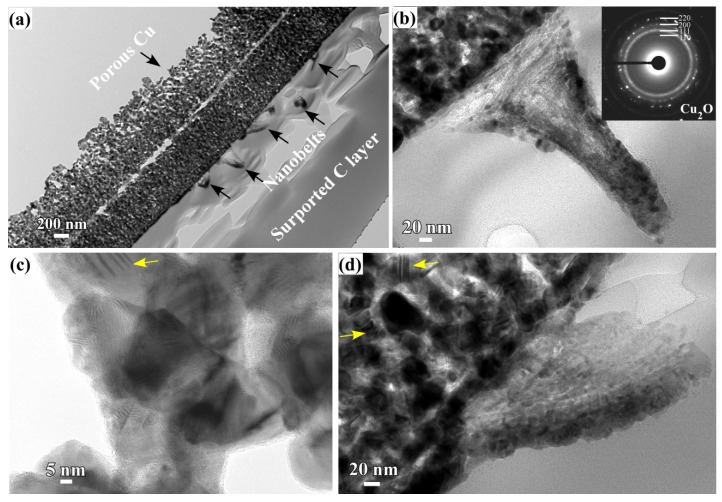
Cross-sectional bright field image (**a**); the magnified image of the root parts of Cu_2_O nanobelts (**b**,**d**) formed on Ti_60_Cu_40_ ribbon dealloyed in 0.03 M HF + 0.01 M PVP solution after immersion of 120 h in ethanol and the high-resolution TEM image (**c**). The inset in b is the SADP.

The roots of the Cu_2_O nanobelts and their interfacial regions seemed growing up at specific sites. In order to know the growth mechanism of Cu_2_O nanobelts, the cross-sectional TEM observation was performed. Several Cu_2_O nanobelts can be seen in the middle parts in Figure 4a. The root of Cu_2_O nanobelts in Figure 4b,d shows the growth against the underlying NPC templates. The heights of the vertical Cu_2_O belts were about 310 nm. The inset selected area diffraction pattern (SADP) of the root indicated that the main composition is Cu_2_O. Cu_2_O mainly existed in a crystalline state since many fringes can be observed in Figure 4c. High-resolution TEM (HRTEM) lattice fringes of Cu_2_O (200) can be confirmed in Figure 4c. There were many lamellar defects (marked by yellow arrows) existing in Figure 4c,d. Those defects were formed during the rearrangement of the Cu adatoms in dealloying, and this facts have been reported before [25,37]. On the other hand, the creation of those defects might be affected by the dealloying temperature, solution chemistry of the dealloying solutions, chemical composition of the precursor alloys, and intermetallic phases etc. In the present experimental conditions, the concentration of HF and PVP might affect the defect density, and surface area of the Cu ligaments. The present combination strategy of immersion of anhydrous ethanol and nanoporous Cu templates (the dealloying solution (0.03 M HF + 0.01 M PVP), precursor alloys (amorphous Ti_60_Cu_40_ alloys without intermetallic phases and defects)) is considered to be favorable for the formation of the Cu_2_O nanobelts. 

### 3.2. 2D Growth of Cu_2_O Nanopetal Networks in Anhydrous Ethanol on NPC Templates from Ti_60_Cu_40_ Ribbons Dealloying in 0.65 M HF and 0.1 M PVP Solution

**Figure 5:**
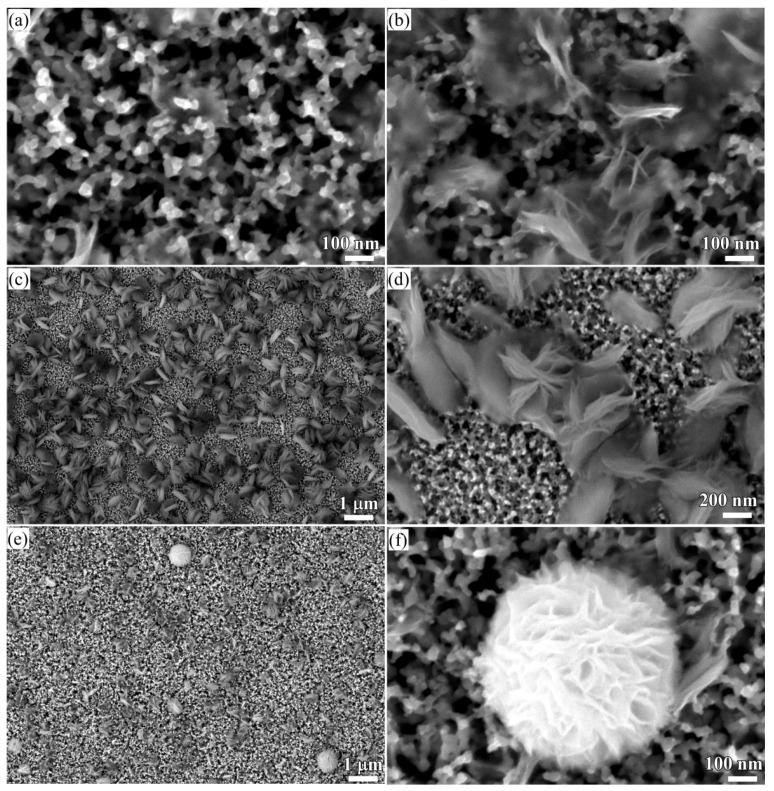
Low-magnificence SEM morphology of Ti_60_Cu_40_ ribbon dealloyed in 0.65 M HF + 0.1 M PVP solution after immersion in anhydrous ethanol for 5 h (**a**,**b**); and for 12 h (**c**,**e**) and corresponding high-magnificence SEM image (**d**,**f**).

**Figure 6:**
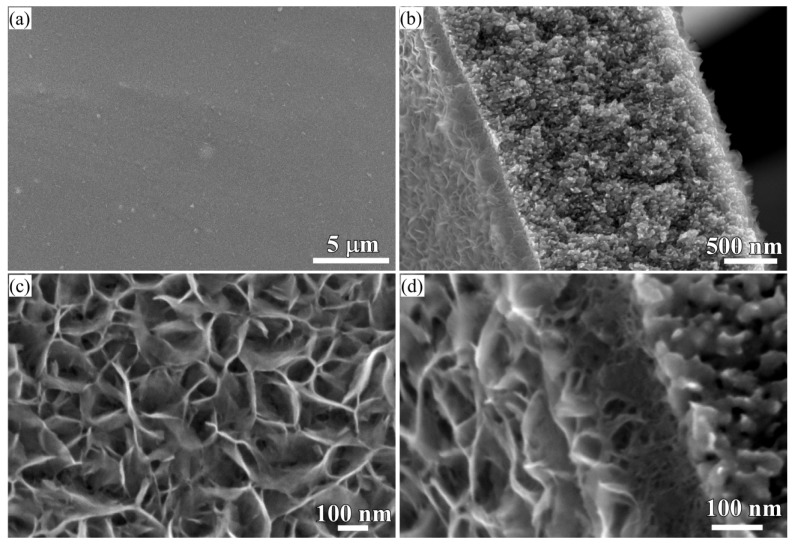
Top-view (**a**) and cross-sectional view (**b**) low-magnificence SEM morphology and their corresponding high magnificence SEM morphology (**c**,**d**) of Ti_60_Cu_40_ ribbon dealloyed in 0.65 M HF + 0.1 M PVP solution after immersion of 120 h in anhydrous ethanol.

**Figure 7:**
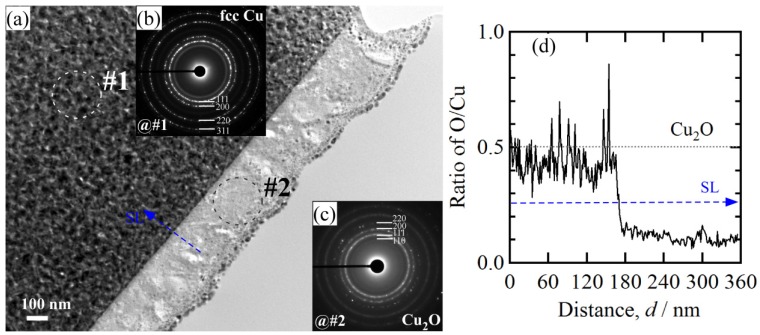
Bright-field TEM images of porous Cu_2_O layer and nanoporous substrate (**a**); SADPs at their corresponding region (**b**,**c**) and the line scan profile (**d**) of the porous layer formed on Ti_60_Cu_40_ ribbon dealloyed in 0.65 M HF + 0.1 M PVP solution after immersion of 120 h in anhydrous ethanol.

**Figure 8:**
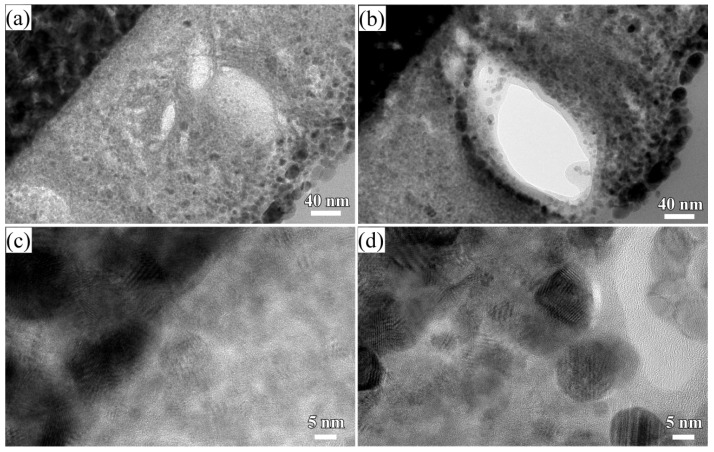
Cross-sectional bright field TEM image (**a**,**b**) at different regions of Cu_2_O porous layer and high-resolution TEM image at the interfacial zone between porous Cu_2_O layer and porous substrate (**c**,**d**).

When some factors (dealloying solutions, immersion time, etc.) are changed, the morphology of Cu_2_O surface nanoarchitectures might be different. The Cu_2_O nanopetals and their networks were formed on the other NPC templates fabricated in the mixture solution of 0.65 M HF and 0.1 M PVP. The NPC structure with a ligament size of 34 nm and a pore size of 25 nm in the surface region was formed. As shown in Figure 5a, some very thin flakes, so-called nanosheets, formed inside the nanopores after immersion of 5 h in anhydrous ethanol. Some wide nanosheets grew and interlaced together in some regions in Figure 5b. After immersion in anhydrous ethanol for 12 h, more and more nanosheets accumulated to form some flower-like nanostructure (hereafter so-called nanopetals). About 65% surface area was covered by nanopetals in Figure 5c. The XRD pattern of nanopetals (Figure 1b) indicates that the nanopetals consisted of Cu_2_O phase. On the other hand, the presence of the diffraction peaks from Cu indicated that the surface Cu_2_O layer was discontinuously distributed. As shown in Figure 5e, the coverage of the Cu_2_O nanpopetals was less than that in Figure 5c. It is interesting that some Cu_2_O nanopetals formed China rose shaped structure. Some small nanosheets were also generated inside the nanopores around the large China rose in Figure 5f. After immersion in anhydrous ethanol for 120 h, the surface of NPC templates were fully covered by the nanopetals in Figure 6a. As indicated by XRD patterns in Figure 1c, the diffraction peaks at 29.5°, 36.3°, 42.4° and 61.4° were assigned to Cu_2_O (110), (111), (200), (220) according to JCPDS card (74-1230). The increase of the intensity and clear splitting of diffraction peaks indicates that the Cu_2_O phase became the dominant one after immersion of 120 h in anhydrous ethanol and the surface Cu_2_O layer covered whole surface. The high magnified top-view SEM morphology in Figure 6c shows that the Cu_2_O nanopetals with very thin thickness interlaced together to form the nanopetals networks. The Cu_2_O nanopetal network was confirmed to be uniform in thickness along the nanoporous Cu template surface and about 300 nm in the cross-sectional SEM observation in Figure 6b. The morphology of the inner structure in Figure 6b and d shows that no large-sized Cu_2_O nanoflakes presented in the nanopores or outside of the Cu ligaments. The low-magnified cross-sectional TEM image in Figure 7a shows that the thickness of the surface Cu_2_O layer was 310 nm. The SADP at Site #1 indicates that the phase of NPC templates is fcc Cu. There are several weak diffraction rings inside the strong diffraction rings of fcc Cu appeared in Figure 7b, which proved that there was small amount of Cu_2_O phase existed in the nanoporous Cu templates after immersion of 120 h in anhydrous ethanol. The SADP at Site #2 is typical of the patterns of Cu_2_O surface layer as shown in Figure 7c. The ratio of O/Cu in Figure 7d was close to 0.5, which is the evidence for the chemical composition of Cu_2_O. The oxygen concentration at the side of NPC templates is not zero indicating the existence of the Cu_2_O. The cross-sectional TEM morphology in Figure 8a,b shows that the characteristics of the Cu_2_O nanopetal networks are porous, and many voids and large pores distributed in the surface Cu_2_O nanopetal networks. Many Cu_2_O crystals with a size of less than 35 nm distributed outside of the surface layer. The HRTEM images at the interface region of Cu_2_O/NPC shows that small-sized.

**Figure 9:**
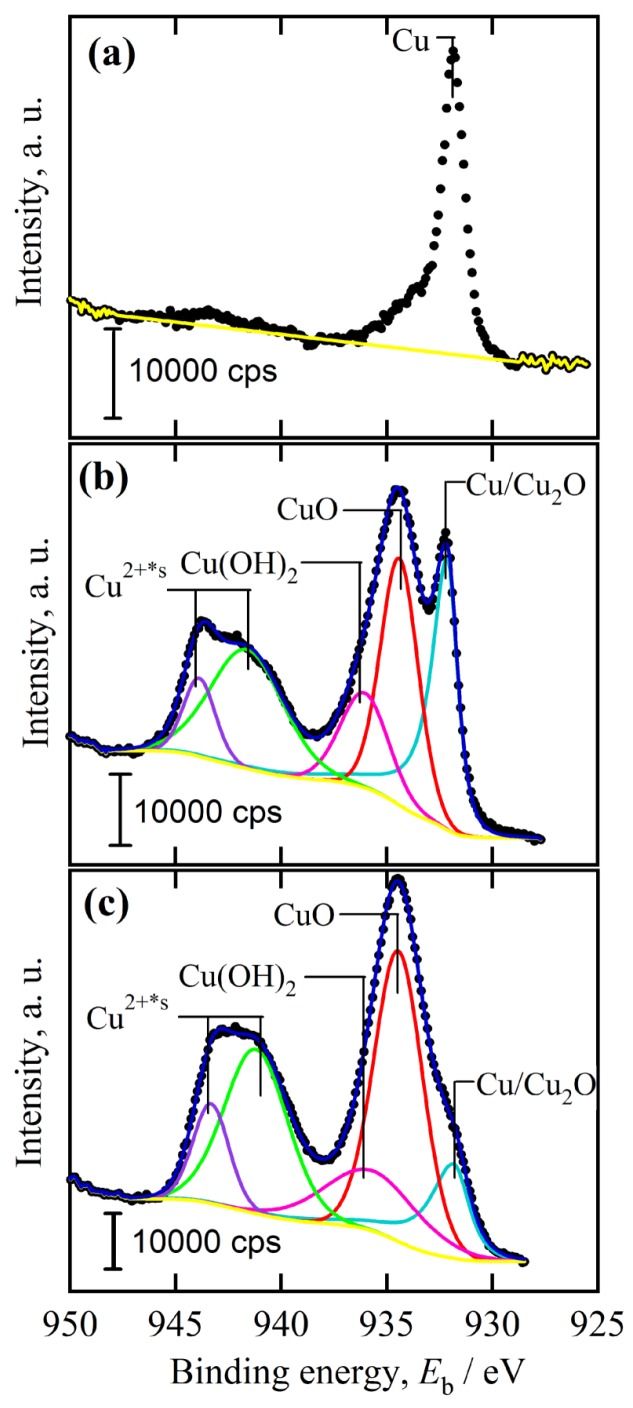
XPS spectra of Cu 2p of Ti_60_Cu_40_ ribbon after dealloying in 0.03 M HF + 0.01 M PVP solution (**a**); after immersion of 120 h in anhydrous ethanol (**b**); XPS spectra of Cu 2p of Ti_60_Cu_40_ ribbon dealloyed in 0.65 M HF + 0.1 M PVP solution after immersion of 120 h in anhydrous ethanol (**c**).

Cu_2_O particles are very finely crystalline from the regular lattice fringes in Figure 8c. The HRTEM images at the outside region of Cu_2_O surface layer exhibited that there are many Cu_2_O crystals with a particle size of about 10–18 nm distributed. The interpanal distance between the adjacent fringes was confirmed to be about 1.83 nm for 10 stacks of the lattice panels, which corresponded to Cu (200). The chemical bonding state of the nanoporous Cu templates, Cu_2_O nanobelts and Cu_2_O nanopetal networks after immersion in anhydrous ethanol for 120 h is shown in Figure 9. As shown in Figure 9a, the Cu 2p peak at 931.9 eV was corresponded to the metallic Cu before immersion in ethanol. The Cu^2+^ has mainly d^9^ character, while the Cu^+^ is expected to have a full 3d shell. After nanoporous Cu templates from Ti_60_Cu_40_ alloys in a mixture solution of 0.03 M HF and 0.01 M PVP were immersed in anhydrous ethanol for 120 h, the position of Cu/Cu_2_O peak shifted to 932.2 eV indicating the increase of the Cu_2_O nanobelts on the surface. The appearance of Cu 2p_3/2_ peak at 934.4 eV and Cu 2p_1/2_ peak at 936.1 eV reflects the oxidation state of Cu compounds with localized valence d orbitals due to the different energies of the photoelectrons [38]. The symbol, Cu^2+*s^, stands for the satellite peaks of Cu 2p. Two shakeup satellite peaks at 941.8 and at 944.1 eV were assigned to bivalent copper. After NPC templates from Ti_60_Cu_40_ alloys in a mixture solution of 0.65 M HF and 0.1 M PVP were immersed in anhydrous ethanol for 120 h, the shape of the spectra are similar except the small difference in the intensity. As it is evident from the curve-fitted spectra in Figure 9c, the Cu 2p region shows two peaks at 934.5 and at 935.9 eV assigned to Cu^2+^ 2p_3/2_ and 2p_1/2_, respectively, along with two shakeup satellite peaks at 941.7 and 943.5 eV. These satellite signals are attributed to Cu^2+^. These subpeak centering at low binding energy of 931.9 eV was attributable to Cu^+^ species. CuO is considered to be oxidized from the Cu_2_O species by oxygen in the air. The smaller subpeak of Cu_2_O is considered to be resulted from the more oxidation of surface Cu_2_O to form CuO or Cu(OH)_2_. The Cu(OH)_2_ species might form as intermediate oxidization products. These species (CuO, Cu(OH)_2_) are considered to be enriched outside of the Cu ligaments. This hypothesis is established on the basis of the low concentration of CuO and Cu(OH)_2_ species due to the absence of the diffraction peaks of CuO and Cu(OH)_2_ in XRD patterns in Figure 1. The present combination of immersion of anhydrous ethanol and finer NPC templates from Ti_60_Cu_40_ alloy in the dealloying solution (0.65 M HF + 0.1 M PVP), precursor alloys is considered to be favorable for the formation of the Cu_2_O nanopetals and nanopetal networks. Meanwhile, the higher concentration of PVP led to the random growth of Cu_2_O nanostructures on the basis of the data presented in Section 3.1 and Section 3.2. The present combination of dealloying solution (higher PVP and HF concentration) and immersion in anhydrous ethanol led to the formation of the Cu_2_O layer on NPC templates. The initial microstructure of NPC templates obviously affected the final characteristics of the surface Cu_2_O products.

### 3.3. Synergistic Effect of Initial Microstructure of NPC Templates and Stabilizing Agent of Ethanol Molecule on the Shapes of Cu_2_O Nanostructure

**Figure 10:**
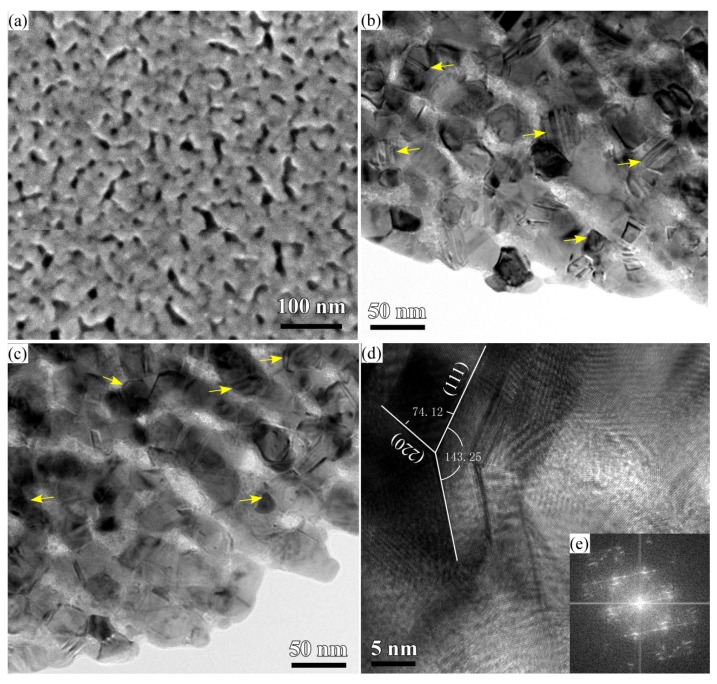
SEM morphology (**a**);TEM BFI image of nanoporous copper fabricated from Ti_60_Cu_40_ ribbon dealloyed in 0.65 M HF + 0.1 M PVP solution (**b**,**c**); the high-resolution TEM image at the site where the twin boundaries exists (**d**) and corresponding FFT pattern (**e**).

The surface SEM morphology of the nanoporous Cu from Ti_60_Cu_40_ alloy in 0.65 M HF and 0.1 M PVP via dealloying in Figure 10a shows that the average pore size was about 8 nm and the average ligament size was about 15 nm. As has been reported, the introduction of PVP into HF solution helps restricting the diffusion and the rearrangement scale of Cu adatoms and reducing the nanopores and ligaments of nanoporous Cu [34]. However, the restricted nucleation and growth of the Cu crystals might cause the increase of the defects, such as twin boundary, stack faults and/or dislocations, etc. Many lamellar defects, stacking faults and other types of defects formed during the dealloying and pileup of the Cu adatoms in Figure 10 b,c. The twin boundary planar defects came from twinning in face-centered cubic structured metallic nanocrystals. HRTEM and fast Fourier transform (FFT) electron diffraction pattern in Figure 10d,e show the dominant features of the lengthwise twins and stacking faults in nanoporous Cu. The distribution density of these kinds of defects was estimated to be 4.20 × 10^17^ m^−2^ for nanoporous Cu dealloyingTi_60_Cu_40_ alloy in 0.03 M HF and 0.01 M PVP and 7.18 × 10^17^ m^−2^ for nanoporous Cu dealloyingTi_60_Cu_40_ alloy in 0.65 M HF and 0.1 M PVP. The additive PVP concentration and the concentration of HF solution affected the final nanoporous microstructure and the density of the defects. On the other hand, the anhydrous ethanol also played a key role of the formation of the nanobelts and nanopetals. The H_2_O molecule as reactant helps the formation of Cu_2_O nanostructures, and ethanol molecule functionalized as a stabilizing or capping reagent for inhibiting the growth of metal nanoparticles [39,40,41]. The ethanol molecule readily adsorb on the Cu surface or Cu_2_O surface, then form intermediate alkoxy species, and further decomposite to create the reductive environment and finally recombines to recover ethanol molecule. The random growth of Cu_2_O nanoarchitectures has been suppressed by the preferential adsorption of ethanol molecule [42]. Many defects (i.e., twin boundary and kinks at the edge of ligaments) in the NPC ligaments in Figure 6 and Figure 10 served as initial sites for the ionization. It is believed that the highly localized reduction of the constrained ions might be responsible for the formation of the flat, highly anisotropic shape, like nanosheet or nanopetal shape. The Cu_2_O compounds preferentially form when solution pH is between 8 and 10, and the alkaline solution enhanced the reduction reaction of cupric (Cu^2+^) cations to cuprous (Cu^+^) cations [43,44]. The formation of Cu_2_O nanostructure obeys the route of Cu_defect_→ionized Cu^+^→partial oxidized Cu^2+^→intermediate Cu(OH)_2_→preferential reduced Cu^+^. The formation of the Cu_2_O nanoarchitectures is affected by curvature of Cu ligaments, the density of defects of nanoporous Cu templates, diffusion of Cu adatoms and Cu^+^ ions and reactions between Cu^+^ and active oxygen atoms. Since the ligaments less than 31 nm exhibit saddle point-like features with highly non-uniform values of the mean curvature, the ionized Cu adatoms at the defects is expected to diffuse predominately by local transport on the scale of the ligament length (order of 10 nm) rather than by long-range transport to the outer surface (up to hundreds of microns) [45]. The increase in the defect density and the decrease of nanopores and ligaments in size certainly resulted in the increase of the nucleation sites and the decrease of the diffusion distance for Cu_2_O nuclei. As shown in the SEM morphology in Figure 2, Figure 5 and Figure 6 and TEM images in Figure 4, Figure 7 and Figure 8, the height of the nanobelts and the thickness of nanopetal networks were almost remain constant after immersion of 24 h similar with the size after 120 h. The nucleation reaction of Cu_2_O is thus regarded as the rate-determining step. Therefore, the increase of the defect density in NPC templates from Ti_60_Cu_40_ alloy in 0.65 M HF and 0.1 M PVP are the most important factor for the formation of the nanopetal networks with increase in the immersion time in anhydrous ethanol. Under the synergistic effect of ethanol molecule and the microstructure of NPC templates, two types of the Cu_2_O nanoarchitectures (nanobelts and nanopetal networks) finally achieved in the present conditions. The present Cu_2_O nanoarchitectures with large active surface area, especially nanopetal networks, have great potential for the DNA biosensors of HBV [46], water splitting for mass hydrogen production [47], catalysis for ethanol/methanol production [48], gas sensor of NO_2_/alcohol/gas oil [49], and lithium ion batteries [50], etc.

## 4. Conclusions

NPC templates with a pore size of 7.8 and 18 nm have been prepared via dealloying amorphous Ti_60_Cu_40_ ribbons in a mixture solution of HF and PVP with different concentrations. Cu_2_O nanoarchitectures have been fabricated via immersion nanoporous copper templates in anhydrous ethanol. Both water molecule reactant acting as OH^−^ reservoir and the ethanol molecule serving as stabilizing or capping reagent for inhibiting the random growth of Cu_2_O, played a role of the formation of two dimensional Cu_2_O nanoarchitectures. Cu_2_O nanobelts are preferred to form in anhydrous ethanol on the NPC templates from Ti_60_Cu_40_ ribbons dealloying in 0.03 M HF and 0.01 M PVP solution, and Cu_2_O nanopetals are tended to grow in anhydrous ethanol from the NPC templates from Ti_60_Cu_40_ ribbons dealloying in 0.65 M HF and 0.1 M PVP solution. With increasing the immersion time in anhydrous ethanol, Cu_2_O nanopetals united together to create porous networks about 310 nm in thickness. The defect sites (i.e., lamellar defects, twin boundary) on the nanoporous Cu ligaments preferentially tended to serve as nucleation sites for Cu_2_O nanocrystals. The different shapes of Cu_2_O nanoarchitectures are affected by the morphology of the initial microstructure of NPC templates, the density of the initial nucleation sites and the solution chemistry of the dealloying solutions.

## References

[1] Kim M.H., Lim B., Lee E.P., Xia Y.N. (2008). Polyol synthesis of Cu_2_O nanoparticles: Use of chloride to promote the formation of a cubic morphology. J. Mater. Chem..

[2] Chen Z.Z., Shi E.W., Zheng Y.Q., Li W.J., Xiao B., Zhuang J.Y. (2003). Growth of hex-pod-like Cu_2_O whisker under hydrothermal conditions. J. Cryst. Growth.

[3] Xu H.L., Wang W.Z., Zhu W. (2006). Shape evolution and size-controllable synthesis of Cu_2_O octahedra and their morphology-dependent photocatalytic properties. J. Phys. Chem. B.

[4] Luo Y.S., Tu Y.C., Ren Q.F., Dai X.J., Xing L.L., Li J.L. (2009). Surfactant-free fabrication of Cu_2_O nanosheets from Cu colloids and their tunable optical properties. J. Solid State Chem..

[5] Cao M.H., Hu C.W., Wang Y.H., Guo Y.H., Guo C.X., Wang E.B. (2003). A controllable synthetic route to Cu, Cu_2_O, and CuO nanotubes and nanorods. Chem. Commun..

[6] Luo Y.S., Li S.Q., Ren Q.F., Liu J.P., Xing L.L., Wang Y., Yu Y., Jia Z.J., Li J.L. (2007). Facile synthesis of flowerlike Cu_2_O nanoarchitectures by a solution phase route. Cryst. Growth Des..

[7] Xu C., Wang X., Yang L.C., Wu Y.P. (2009). Fabrication of a graphene–cuprous oxide composite. J. Solid State Chem..

[8] Wang D.B., Mo M.S., Yu D.B., Xu L.Q., Li F.Q., Qian Y.T. (2003). Large-scale growth and shape evolution of Cu_2_O cubes. Cryst. Growth Des..

[9] Tan Y.W., Xue X.Y., Peng Q., Zhao H., Wang T.H., Li Y.D. (2007). Controllable fabrication and electrical performance of single crystalline Cu_2_O nanowires with high aspect ratios. Nano Lett..

[10] Kuo C.H., Chen C.H., Huang M.H. (2007). Seed-Mediated synthesis of monodispersed Cu_2_O nanocubes with five different size ranges from 40 to 420 nm. Adv. Funct. Mater..

[11] Gou L.F., Murphy C.J. (2003). Solution-phase synthesis of Cu_2_O nanocubes. Nano Lett..

[12] Wiley B., Sun Y.G., Mayers B., Xia Y.N. (2005). Shape-controlled synthesis of metal nanostructures: The case of silver. Chem. Eur. J..

[13] Xiong Y.J., Xia Y.N. (2007). Shape-controlled synthesis of metal nanostructures: The case of palladium. Adv. Mater..

[14] Sun Y.G., Xia Y.N. (2004). Mechanistic study on the replacement reaction between silver nanostructures and chloroauric acid in aqueous medium. J. Am. Chem. Soc..

[15] Zhang H.G., Zhu Q.S., Zhang Y., Wang Y., Zhao L., Yu B. (2007). One-pot synthesis and hierarchical assembly of hollow Cu_2_O microspheres with nanocrystals-composed porous multishell and their gas-sensing properties. Adv. Funct. Mater..

[16] Xu J.S., Xue D.F. (2007). Five branching growth patterns in the cubic crystal system: A direct observation of cuprous oxide microcrystals. Acta Mater..

[17] Chang Y., Zeng H.C. (2004). Manipulative synthesis of multipod frameworks for self-organization and self-amplification of Cu_2_O microcrystals. Cryst. Growth Des..

[18] Xiong Y.J., Li Z.Q., Zhang R., Xie Y., Yang J., Wu C.Z. (2003). From complex chains to 1D metal oxides: A novel strategy to Cu_2_O nanowires. J. Phys. Chem. B.

[19] Zhang X., Xie Y., Xu F., Liu X.H., Xu D. (2003). Shape-controlled synthesis of submicro-sized cuprous oxide octahedra. Inorg. Chem. Commun..

[20] Zhang H.W., Zhang X., Li H.Y., Qu Z.K., Fan S., Ji M.Y. (2007). Hierarchical growth of Cu_2_O double tower-tip-like nanostructures in water/oil microemulsion. Cryst. Growth Des..

[21] Siegfried M.J., Choi K.S. (2004). Electrochemical crystallization of cuprous oxide with systematic shape evolution. Adv. Mater..

[22] Siegfried M.J., Choi K.S. (2005). Directing the architecture of cuprous oxide crystals during electrochemical growth. Angew. Chem. Int. Ed..

[23] Siegfried M.J., Choi K.S. (2008). Elucidation of an overpotential-limited branching phenomenon observed during the electrocrystallization of cuprous oxide. Angew. Chem. Int. Ed..

[24] Li J., Shi Y., Cai Q., Sun Q.Y., Li H.D., Chen X.H., Wang X.P., Yan Y.J., Vireling E.G. (2008). Patterning of nanostructured cuprous oxide by surfactant-assisted electrochemical deposition. Cryst. Growth Des..

[25] Zhang Z.H., Wang Y., Qi Z., Zhang W.H., Qin J.Y., Frenzel J. (2009). Generalized fabrication of nanoporous metals (Au, Pd, Pt, Ag, and Cu) through chemical dealloying. J. Phys. Chem. C.

[26] Erlebacher J., Aziz M.J., Karma A., Dimitrov N., Sieradzki K. (2001). Evolution of nanoporosity in dealloying. Nature.

[27] Dan Z.H., Qin F.X., Wada T., Yamaura S., Xie G.Q., Sugawara Y., Muto I., Makino A., Hara N. (2013). Nanoporous palladium fabricated from an amorphous Pd_42.5_Cu_30_Ni_7.5_P_20_ precursor and its ethanol electro-oxidation performance. Electrochim. Acta.

[28] Yu J.S., Ding Y., Xu C.X., Inoue A., Sakurai T., Chen M.W. (2008). Nanoporous metals by dealloying multicomponent metallic glasses. Chem. Mater..

[29] Dan Z.H., Qin F.X., Sugawara Y., Muto I., Hara N. (2012). Fabrication of nanoporous copper by dealloying amorphous binary Ti-Cu alloys in hydrofluoric acid solutions. Intermetallics.

[30] Dan Z.H., Qin F.X., Hara N. (2014). Refinement of nanoporous copper: A summary of micro-alloying of Au-group and Pt-group elements. Mater. Trans..

[31] Dan Z.H., Qin F.X., Sugawara Y., Muto I., Hara N. (2012). Bimodal nanoporous nickel prepared by dealloying Ni_38_Mn_62_ alloys. Intermetallics.

[32] Dan Z.H., Qin F.X., Sugawara Y., Muto I., Hara N. (2013). Elaboration of nanoporous copper by modifying surface diffusivity by the minor addition of gold. Microporous Mesoporous Mater..

[33] Dan Z.H., Qin F.X., Yamaura S., Xie G.Q., Makino A., Hara N. (2014). Refinement of nanoporous copper by dealloying MgCuY amorphous alloys in sulfuric acids containing polyvinylpyrrolidone. J. Electrochem. Soc..

[34] Dan Z.H., Qin F.X., Hara N. (2014). Polyvinylpyrrolidone macromolecules function as a diffusion barrier during dealloying. Mater. Chem. Phys..

[35] Kou T.Y., Jin C.H., Zhang C., Sun J.Z., Zhang Z.H. (2012). Nanoporous core–shell Cu@Cu_2_O nanocomposites with superior photocatalytic properties towards the degradation of methyl orange. RSC Adv..

[36] Liu D.Q., Yang Z.B., Wang P., Li F., Wang D.S., He D.Y. (2013). Preparation of 3D nanoporous copper-supported cuprous oxide for high-performance lithium ion battery anodes. Nanoscale.

[37] Liu W.B., Zhang S.C., Li N., Zheng J.W., Xing Y.L. (2011). A facile one-pot route to fabricate nanoporous copper with controlled hierarchical pore size distributions through chemical dealloying of Al–Cu alloy in an alkaline solution. Microporous Mesoporous Mater..

[38] Chanquía C.M., Sapag K., Rodríguez-Castellόn E., Herrero E.R., Eimer G.A. (2010). Nature and location of copper nanospecies in mesoporous molecular sieves. J. Phys. Chem. C.

[39] Li D., McCann J., Matthew T., Xia Y.N. (2004). Photocatalytic deposition of gold nanoparticles on electrospun nanofibers of titania. Chem. Phys. Lett..

[40] Leff D.V., Ohara P.C., Heath J.R., Gelbart W.M. (1995). Thermodynamic control of gold nanocrystal size: Experiment and theory. J. Phys. Chem..

[41] Wiley B.J., Xiong Y.J., Li Z.Y., Yin Y.D., Xia Y.N. (2006). Right bipyramids of silver: A new shape derived from single twinned seeds. Nano Lett..

[42] Bowker M., Madix R.J. (1982). XPS, UPS and thermal desorption studies of alcohol adsorption on Cu(110): II. Higher alcohols. Surf. Sci..

[43] Ko E.S., Choi J., Okamoto K., Tak Y.S., Lee J.Y. (2006). Cu_2_O nanowires in an alumina template: Electrochemical conditions for the synthesis and photoluminescence characteristics. ChemPhysChem.

[44] Choi J.S., Ko E.S., Kang J.W., Tak Y.S., Lee J.Y. (2007). Influence of solution pH on the electrochemical fabrication of functional metal oxides using a nanoporous alumina template. J. Ind. Eng. Chem..

[45] Parida S., Kramer D., Volkert C.A., Rosner H., Erlebacher J., Weissmüller A. (2006). Volume change during the formation of nanoporous gold by dealloying. Phys. Rev. Lett..

[46] Zhu H.T., Wang J.X., Xu G.Y. (2009). Fast Synthesis of Cu_2_O Hollow Microspheres and Their Application in DNA Biosensor of Hepatitis B Virus. Cryst. Growth Des..

[47] Luo J.S., Steier L., Son M.K., Schreier M., Mayer M.T., Grätzel M. (2016). Cu_2_O nanowire photocathodes for efficient and durable solar water splitting. Nano Lett..

[48] Christoforidis K.C., Fornasiero P. (2017). Photocatalytic hydrogen production: A rift into the future energy supply. ChemCatChem.

[49] Zhang J.T., Liu J.F., Peng Q., Wang X., Li Y.D. (2006). Nearly monodisperse Cu_2_O and CuO nanospheres:  Preparation and applications for sensitive gas sensors. Chem. Mater..

[50] Zhang C., Tu J., Huang X., Yuan Y., Chen X., Mao F. (2007). Preparation and electrochemical performances of cubic shape Cu_2_O as anode material for lithium ion batteries. J. Alloy. Compd..

